# Recent progress of Bioinspired Hydrogel-based delivery system for endometrial repair

**DOI:** 10.3389/fbioe.2022.1013217

**Published:** 2022-09-09

**Authors:** Rong Dong, Saihua Ma, Xiaoli Zhao, Baojuan Wang, Mridul Roy, Lu Yao, Tian Xia, Yanting Liu

**Affiliations:** ^1^ First Teaching Hospital of Tianjin University of Traditional Chinese Medicine, National Clinical Research Center for Chinese Medicine Acupuncture and Moxibustion, Tianjin, China; ^2^ Department of Oncology, The First Affiliated Hospital of Xinxiang Medical University, Weihui, China; ^3^ Hemay Zhihui Science and Technology Co. Ltd, Tianjin, China

**Keywords:** bioinspired, hydrogel, uterine, endometrial, reproductive

## Abstract

Endometrial injury is the main fact leading to infertility. Current treatments of endometrial injury present many problems, such as unable to achieve desired effects due to low retention and the inherent potential risk of injury. Besides, it is important to the development of bioinspired material that can mimic the natural tissue and possess native tissue topography. Hydrogel is a kind of bioinspired superhydrophilic materials with unique characteristics, such as excellent biocompatibility, biodegradability, porosity, swelling, and cross-linkage. These unique physiochemical properties of bioinspired hydrogels enable their promising application as novel delivery platform and alternative therapies for endometrial injury. In this mini review, we summarize the recent advances in bioinispred hydrogel-based delivery system for endometrial repair, including as a post-operative physical barrier and therapeutic delivery system. In addition, present status, limitations, and future perspectives are also discussed.

## 1 Introduction

Endometrium is the innermost layer of the uterus. This layer comprises epithelial and stroma components that are responsible for cell proliferation, differentiation, and shedding—which is deterministic of a woman’s viability for embryo implantation (Lessey, 2000). The normal endometrium can repair and regenerate itself and plays a pivotal role in female physiology and reproductive function. However, severe endocrine disorders, intrauterine infection and intrauterine surgery can lead to endometrial injury. When the uterine cavity lacks endometrial coverage, fibrosis, scarring and adhesion may occur, which result in decreased reception, loss, or reduced regenerative ability (Deans and Abbott, 2010; Liao et al., 2021; Lv et al., 2021), and those are the common cause of infertility worldwide (Ma et al., 2021).

Traditionally, there are three main treatments for endometrium repair, including surgical separation of the adherent endometrium, estrogen and cytokines therapy, and stem cell therapy (Yu et al., 2008; Evans-Hoeker and Young, 2014; Hooker et al., 2016)). For surgical treatment, the re-adhesion rate is high. For drug and stem cell therapy, the retention to the sites of endometrium is quite low (Abomaray et al., 2016; Liu et al., 2016; Xie et al., 2017; Mao et al., 2020). Moreover, the stem cells easily flow with blood and body fluids, making them difficult to be delivered to the damaged endometrium (Ma et al., 2021). Contrary, recent study shed light on the potential efficiency of constructed uterus-inspired niche for the efficient developmental events during the early stage of organogenesis (Gu et al., 2022). Knowledge of the above assertion prompts a need for a bio-material to provide a favorable environment for stem cell adhesion, controlled drug release and post-operation anti-adhesive barrier for effective repair of the endometrium.

Hydrogel is a kind of bioinspired superhydrophilic materials, which have similar compositional and structural features with natural tissues (Chen et al., 2021). Their polymeric three-dimentional (3D) network enables them to absorb and retain a significant amount of water. The water content of hydrogel can be as high as 90% (Zhang et al., 2019). Moreover, bioinspired hydrogel possesses excellent properties, such as high water absorbability, biocompatibility, low interfacial tension and degradability (Table 1). What’s more, the above mentional properties of bioinspired hydrogel can be tuned both physically and chemically, gaining great interests in a variety of applications such as tissue engineering, drug release, and 3D cell culture (Lim et al., 2014). All in all, hydrogel is a perfect platform for encapsulating and delivery of novel intrauterine therapies (Ma et al., 2018; Tang et al., 2018; Marycz et al., 2019), and serve as a promising biomaterial for endometrium repair (Townsend et al., 2019).

**TABLE 1:** The physiochemical properties of hydrogels.

Characteristic	Physiochemical property	Function
1. Swelling property	The presence of hydrophilic, carboxyl and hydroxyl groups	Serve as therapeutic cargo or scaffold for delivery
2. Pore containing structure	Incorporation of porogens	Provide rapid cellular infiltration with maintaining structural integrity
3. Self-healing	Polymeric networks within the hydrogel matrix are mediated by either weak sacrificial noncovalent ionic, hydrogen or hydrophobic interactions or dynamic chemical covalent bonds	Repair structural damages to recover original functions applied for wound healing, tissue engineering, surface coating, or drug/cell delivery
4. Biocompatibility	Incorporation of polymeric substances, the internal water content and appropriate viscosity	Helps adhere to the injured site, often used as artificial ECM that mimics the tissue environment

## 2 Application of bioinspired hydrogel in endometrium injury

Hydrogels have unique properties that make them enable for application in regenerative medicine. These properties include excellent biocompatibility and biodegradability, strong water-binding capacity, which enables them to double their size while swelling, an ability to incorporate therapeutic components for controlled release as well as, the ability to encapsulate seed cells at optimum physiological conditions (Wang et al., 2021). For endometrial injury, hydrogels are applied as a physical anti-adhesion barrier and as a delivery mechanism for therapeutics. The physical and biological properties of hydrogels enable them to influence the uterine microenvironment, biological behaviors, proliferation and angiogenesis. Swelling property makes hydrogel capable of compressing the bleeding point. The interactions between blood cells and the charged amino groups on the hydrogels confer aggregation, adhesion and blood clotting (Gu et al., 2010; Wen et al., 2016). The amino group, in association with the hydroxyl group of hydrogels also reacts with the oxygen free radicals to reduce the reactive oxygen-mediated damage of endometrium (Castro Marin et al., 2019; Wei et al., 2019). In addition, hydrogels can inhibit the release of TNF-α and IL-6 and increase the production of IL-8. These IL-8 induce an anti-inflammatory response on one hand and also combine with the neutral granulocytes surface receptors CXCR1/2 to produce active components against bacteria.

### 2.1 Application of bioinspired hydrogel as a post operative Physical Barrier

Conventional post-transcervical resection of adhesion (TCRA) anti-adhesion physical barriers such as intrauterine devices (IUD) and Foley catheter balloons pose the risk of recurring adhesion owing to a limited surface area (Bhandari et al., 2015; Chi et al., 2018). This implies that they are unable to cover the anterior and posterior walls of the uterus effectively. Another underlying issue is the risk of infection during their removal and their inability to promote pregnancy. Biomedical researches indicate the application of optimized hydrogels to be efficient in this regard (Xiao et al., 2015). Hyaluronic acid (HA) hydrogel is biocompatible, biodegradable as well as non-toxic to the cells and tissues. In fact, HA hydrogels were proven to reduce the risk of the reformation of intrauterine adhesions (IUAs) in women with an endometrial injury who undergo resection surgery (Can et al., 2018; Lee et al., 2020a; Tafti et al., 2021). However, studies found that hydrogel combined treatment strategy with the insertion of a urinary catheter or IUD had a more satisfactory effect in regaining an adhesion-free uterine cavity (Xiao et al., 2015; Li et al., 2019; Pabuccu et al., 2019). A meta-analysis indicated that Hyaluronic acid (HA) hydrogel platforms and chitosan platforms used after TCRA facilitate excellent tissue repair, and reduce adhesion recurrence but did not affect on postoperative pregnancy rate among patients (Fei et al., 2019). The latter is consistent with the research findings of Mao et al. (2020) which suggest HA hydrogels could enhance endometrial receptivity and clinical pregnancy rates in moderate IUA patients. Although many studies proved IUD combined with hydrogels reduce IUA recurrence, in some cases, the pregnancy rate remains lower in these patients. Furthermore, due to rapid degradation, it cannot stay for a prolonged duration inside the uterine cavity (Azumaguchi et al., 2019). Also, following hysteroscopic adhesiolysis, auto-cross-linked HA gel was found inefficient to reduce the recurrence rate of adhesion (Zhou et al., 2021b). This can be explained by, the use of concurrent adjuvant therapy which can mask the beneficial effect of hydrogels in reducing adhesion recurrence. Furthermore, the severity of pre-existing adhesions and the types of the gel used in different studies were also different. Whatever, the results suggesting the necessity of in-depth studies to explore the appropriate application of hydrogels to improve patients outcomes.

### 2.2 Application of bioinspired hydrogel as a Therapeutic Delivery System

Novel treatment emerged as a postoperative mechanism after TCRA and hysteroscopic adhesiolysis to facilitate the repair and regeneration of the endometrium and reduce the recurrence of adhesion. In the case of drug treatment for endometrial injuries, drugs may be administered via oral means, transvaginal or intravenous injections. Albeit, complications resulting from these drug administration methods, such as damage to liver and blood tissues (Lin et al., 2020), claim the requirement of other efficient modes of delivery of the drugs to the injured site. Also, stem therapy options facilitated the possibility of substitution or replacement of injured cells to aid in endometrium regeneration (Tan et al., 2016; Gan et al., 2017; Yin et al., 2019). Both therapies result in limited therapeutic effect due to the low retention to the sites of injury (Abomaray et al., 2016). For optimum therapeutic effect, the materials (drug or 3D stem cells) should be delivered and retained to the injured site to prevent bacterial infection. Hydrogel used in endometrial regeneration and repair is highly biocompatible, biodegradable and has a porous structure for encapsulation as well as the sustained release of materials. Therapeutic materials to be delivered include; estrogen, cytokines, stem cells and exosomes.

#### 2.2.1 Application of Hydrogel as a Drug Delivery System

**FIGURE 1:**
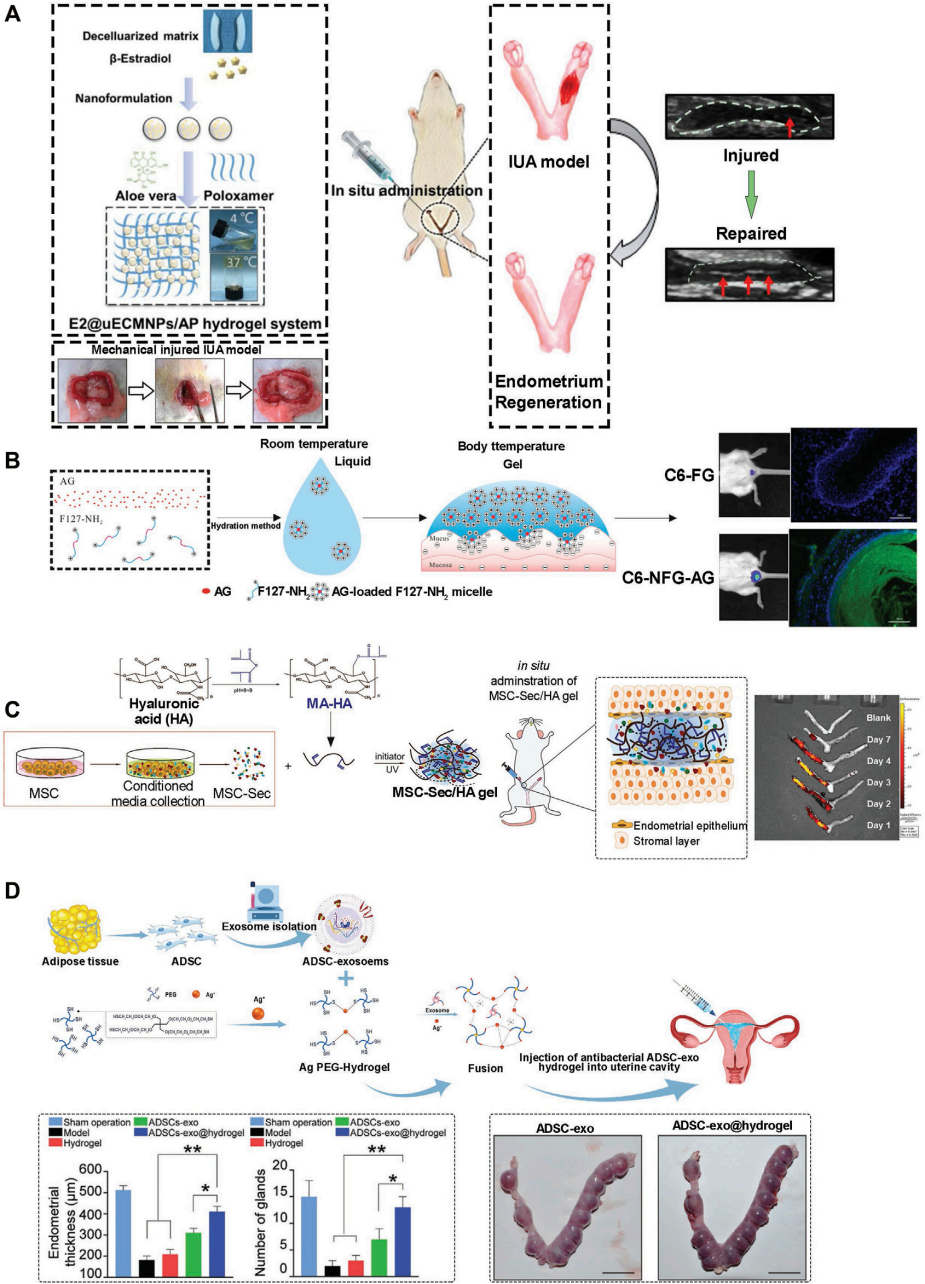
Application of hydrogel in endometrium injury. **(A)** E2@uECMNPs/AP hydrogel system for B-estradiol. Following establishment of rat intrauterine adhesion (IUA) model, and treatment the theragnostic ultrasound test was employed to compare the images of the injured IUA with or without E2@uECMNPs treatment (Yao et al., 2020). **(B)** Poloxamer 407 (F127)-based *in situ* hydrogel for the delivery of acetate gossypol (AG) as a model drug. Intravaginal retention of NFG (acetate gossypol-loaded aminated poloxamer 407-based temperature-sensitive hydrogel) and FG (F127 gel) was evaluated. Photographs and fluorescence microscopy showed NFG prolonged intravaginal residence (Ci et al., 2017). **(C)** HA-Hydrogel integrated with mesenchymal stem cell (MSC) to treat endometrial injury in a rat model. Schematic showing the synthesis of MSC-Sec-loaded, crosslinked HA gel. MSC-Sec/HA gel was injected in rodent model of endometrium injury and *ex vivo* fluorescent imaging of rat uteri showed the crosslinked HA can stay in the uterine cavity for roughly two estrous cycles (Liu et al., 2019). **(D)** Schematic overview of the development of an exosome secreted by adipose-derived stem cell (ADSC-exo) hydrogel for endometrial regeneration. Increase of endothelial thickness with a concomitant increase of gland numbers affirmed the ability of ADSC-exo hydrogel to promote tissue regeneration. Beside, no significantly higher pregnancy and implantation rates in the ADSC-exo and ADSC-exo hydrogel groups evidant normal endometrial formation and function (Lin et al., 2021).

Hydrogel presents distinctive properties that interest its application for drug delivery in the repair and regeneration of endometrial injury. First, the porous structure enables the loading and control release of treatment drugs. Drugs such as β-estradiol can be delivered by hydrogel scaffolds for various purposes including endometrial regeneration (Zhang et al., 2017). Furthermore, the use of stimuli-responsive hydrogels brings many possibilities to drug delivery systems. Poloxamer hydrogel is a thermosensitive hydrogel with better fluidity. When optimized via a polycondensation reaction into heparin-modified poloxamer hydrogel, the half-life of its growth factors is extended and fluidity improved. It covers the injured site completely and perfectly becomes solid at equilibrium temperature (normal body temperature). This prevents bacterial infection and loss of the drug. Thus, injecting the thermosensitive hydrogel (E2-HPhydrogel) with the encapsulation of 17β-estradiol into the injured uterine cavity eliminates the weaknesses of exogenous administration of 17β-estradiol including water solubility, limited half-life time, and low concentrations at the injured area (Baghersad et al., 2018). Hormones such as estrogen, cytokine, exosomes, etc. could also be delivered efficiently and effectively while capitalizing on hydrophilic polymer chain linkage in hydrogels. With regards to estrogen release, the application of poloxamer hydrogel as a carrier does not prolong retention time. Aloe has traits that make it an ideal organic component to mix with poloxamer to form a more biologically friendly thermosensitive hydrogel system (Baghersad et al., 2018; Yao et al., 2020). Aloe-poloxamer (AP) hybrid hydrogel has been fabricated for treating endometrial injury and achieved a better therapeutic effect with a prolonged retention time (Figure 1A). Yao et al. designed a nano-composite aloe/poloxamer hydrogel containing E2 with an additional benefit to enhance the therapeutic effects of estrogen on endometrial regeneration by upregulating estrogen receptors, reducing the likelihood that high-dose estrogen would increase the risk of thrombosis and malignancy. Hydrogel as drug delivery system may be applied as *in situ* vaginal administration in a low viscous form at room temperature, further functionalization, such as with amino group, of poloxamer-based hydrogel prolong intravaginal residence (Figure 1B). Studies suggested that the large surface area and high vascularized nature of the vaginal area enable excellent drug absorption (Liu et al., 2017b; Ci et al., 2017). Again, vaginal administration has no first-pass effect and has low enzyme activity; therefore, drugs can perform a localized activity, and can also enter the systemic circulation. When thermosensitive hydrogel is administered vaginally, the hydrogel spreads rapidly into the folded area of the vaginal mucosa and forms a gel at body temperature (Andrzejewska et al., 2019). Drug delivery system through injectable hydrogels, as another option, provides other benefits including shortened duration of treatment, decreased risk of infection and prevention of scarring (Asai et al., 2012).

#### 2.2.2 Application of bioinspired Hydrogel as a Three-dimensional Cell Delivery and Culture System

Recent advances in the treatment of endometrium injury suggest cell therapy as a promising alternative to drug therapy. For example, Yi et al. stated that similar to normal rats, rats with injured uterus achieve nearly complete recovery, following treatment with uterus-derived extracellular matrix and seeded chorionic villi mesenchymal stem cells-combined reconstructable uterus-derived materials (RUMs) (Yi et al., 2022). Transplanting Mesenchymal Stem Cells (MSC) via hydrogel as a delivery system showed good potential for endometrial repair (Figure 1C) (Nelson et al., 2009). There have been several sources of stem cells that have been proposed for tissue regeneration. Bone marrow-derived MSC (BM-MSC) has been the most promising cell source of regeneration due to ease in acquisition, self-renewal ability, multi potential differentiation and weak immunogenicity (Ding et al., 2014; Zhou et al., 2021a). Other sources of stem cells include; Human umbilical cord mesenchymal stem cell (UCMSC), Endometrial stromal cell (EMSC), Endometrial perivascular cell (ENPSC) and Bone mononuclear cell (BMNC). Interestingly, hydrogels can be designed as culture systems with a tunable stiffness that encompasses a physiological range owing to their distinguishable properties. Normally hydrogels used for such applications have high-water content, excellent porosity and soft consistency. Also, such hydrogels can closely simulate natural living tissue. Collagen, a natural hydrogel, is widely used in conjunction with the BM-MSC for healing wounds and tissue regeneration as evidence shown in a research study by Ding et al. (Liu et al., 2017a). Again, other synthetic hydrogels used in conjunction with the stem cells include Pluronic F-127 (PF-127). However, it has been found that these synthetic models may have toxic side effects on the cells and thus, there is a need for antioxidants within the scaffold. Commonly, Vitamin C reduces the cytotoxic effect of PF127, and promotes the survival and growth of cells by influencing ECM and collagen homeostasis, exerting anti-inflammatory functions by downregulating the secretion of proinflammatory cytokines such as TNF-a and interleukin-6 (IL-6). Another high-quality carrier for BM-MSC is the photo-crosslinked PRP hydrogel (HNPRP) (Wenbo et al., 2020). The material can be quickly prepared *in situ* to form a strong scaffold and was demonstrated to achieve controlled release of growth factors and reduce tissue adhesion. All the above exogenous substances have potential safety problems such as immunoreaction risk. As such other research studies choose endogenous stem cell migration (Han et al., 2016). *In situ* delivery of stromal cell derivative-1α (SDF-1α) in a controlled release could accelerate the regeneration of multiple tissues.

#### 2.2.3 Application of bioinspired Hydrogel as Exosome Delivery System

Exosomes are extracellular vesicles with a size range of 40–160 nm in diameter. Exosomes, both on their surfaces or inside of them, mainly contain cargo, such as lipids, proteins and nucleic acids, including DNA, mRNA and miRNA (Mathivanan et al., 2010; D'Asti et al., 2012). Under normal physiological conditions, exosomes can originate from endothelial cells, immune cells, tumor cells and mesenchymal stem cells (MSCs) and mimic the extracellular matrix or act as regulators of intercellular communication and immune response (Huang et al., 2021). Exosomes from MSCs are capable of functions similar to MSCs including tissue regeneration and repair (Askenase, 2020). However, exosomes showed several advantages over MSCs, including reduced risk of immune rejection and malignant growth, longer stability and readily circulatory capacity through capillaries (Wang et al., 2021). Owing to all these advantageous characteristics which make exosomes a current research hotspot, the use of purified exosomes can be limited by their rapid clearance from the host after being absorbed by the endothelial system. This limitation can be overcome by hydrogels that can protect exosomes acting as a carrier and delivery depot at the target site to achieve well-regulated cellular secretions more stable therapeutic effect (Figure 1D). Because of the unique physiochemical characteristic of hydrogel and the ability to controlled-release its embedded molecule, encapsulation of exosomes with hydrogel provide an outstanding candidate in plenty of treatments including bone and cartilage regeneration, cardiovascular diseases, spinal cord injury, periodontal and corneal repairs (Xie et al., 2022). Exosome has immense potential of treating injured endometrium by regulating EMT (Yao et al., 2019; Feng et al., 2020), miRNA (Tan et al., 2020; Shi et al., 2021), proliferation (Lv et al., 2020) and endometrium angiogenesis (Zhang et al., 2022). However, all these effects are achieved *via* the suboptimal approach of direct injection which restricts the activity of exosomes by local tissue irritation or reduced self-life. The ideal repair of endometrium by exosomes can be achieved by using the biocompatibility and physically tunable properties of hydrogels. The exosome-hydrogel system proved to be a safe, noninvasive and convenient method for repairing injured endometrium and can exert excellent effects by promoting angiogenesis, inhibiting local tissue fibrosis and increasing endometrial receptivity (Lin et al., 2021). Although the results were promising, the exosome-hydrogel also has the disadvantage that the bioscaffolds lack the native tissue topography to mimic the natural tissues.

## 3 Present status and future outlook

Although the damaged endometrium cells can be self-healing and self-regenerated in patients, treatment options such as drugs (estrogen, cytokines, 17β-estradiol, cytokines, etc.) and stem cell therapy aid in the repair and regenerative process. However, it has been found that these therapies usually have low retention to the sites of injury and thus, the desired therapeutic effect is not achieved (Zhao et al., 2021). Bioinspired hydrogel is a 3D hydrophilic polymer with excellent biocompatibility and biodegradability features. It have been identified as having the ability to transport and deliver these therapeutics to the sites of injury owing to their porous, biodegradable and biocompatible features. Coupled with its other distinct features, the material finds varied applications in endometrial regeneration and repair. It serves as an excellent anti-adhesion physical barrier, drug delivery system, three-dimensional cell delivery, and exosome delivery system. While multiresponsive hydrogels that respond to redox, pH and temperature are being proved effective in physiological environments (Lou et al., 2015). Hence, the development of multiresponsive hydrogels to adapt to the intrauterine environment is a future meaningful subject. In addition to physiologically relevant stimuli, the response of these polymers needs to measure in the presence of prevalent biomedical external stimuli such as magnetic field and UV light. To develop an innovative delivery system with excellent biocompatibility, the future direction of research needs compilation of the responsive properties of hydrogels in nearly every conceivable arrangement and minimize the synthesis complexity of these multi-responsive hydrogels. Hydrogels may be optimized to be sensitive to physiological environmental changes to facilitate their specificity and controlled release of treatment. This demonstrates the potential benefits of the encapsulation of cells or other therapeutic substances and underlines the importance of hydrogels in endometrial repair and regeneration. However, to achieve desired and effective therapeutic effects, it is recommended that future studies consider the extending application of multiresponsive hydrogel delivery system to endometrial injury treatment.

The hydrophobic and electrostatic interactions in hydrogels aid the supramolecular assembly of amphiphiles that hold a large amount of water. As a result, hydrogels are potential drug reservoirs and capable of maintaining slow and sustained release. Utilizing this property, researches entrapped two or more drugs in a biodegradable hydrogel to produce a synergistic effect against different ailments including cancer (Medatwal et al., 2020). Therefore, optimization of hydrogels by using rational molecular design to carry multiple drugs for emdometrium regeneration should be considered as this would improve the overall repair and regenerative process of the injured endometrium (An et al., 2021). This will aid in resolving infertility in patients.

Gene editing technology is considered as the driver of modifying genes in fundamental research and facilitating gene therapies in clinical developments. The invention of CRISPR (clustered regularly interspaced short palindromic repeats) Cas (CRISPR-associated) toolbox is a remarkable breakthrough for gene editing. The system contains a complex of the guide RNA (gRNA) that recognizes its complementary target DNA and the Cas nuclease that cut the dsDNA (Knott and Doudna, 2018; Es et al., 2019). Though the system is simple and flexible, the efficiency for targeted delivery of both gRNA and Cas9 needs thoughtful consideration (Lindsay-Mosher and Su, 2016). Biocompatible DNA hydrogels, harboring the crosslinked structure and programmable property, can be harnessed for the targeted transport of CRISPR biomaterials. Although gene therapy is not common for IUA, future research may be directed to using a CRISPR-DNA hydrogel system for the improvement of endometrium recovery. In recent years, bioprinting has gained significant attention not only for the fabrication of biomimetic tissue constructs but also in pharmaceuticals, improving drug screening, disease research, and controlled drug-delivery systems. Hydrogels, being the most common bioink for bioprinting, provide a supportive hydrated environment for cells and preserve the shape of printed materials (Wang et al., 2017). Through various crosslinking, hydrogels are capable of achieving different tissue engineering applications as bioinks and support baths (Zhou et al., 2022). Hydrogel bioinks that can respond to external stimuli, such as gelatin methacrylate (GelMA) polymer, photo-cross linked HA-hydrogels or polyethylene glycol and alginate hydrogel, proved to be outstanding materials for 3D bioprinting of human organoid including vascularized soft tissues, cardiac muscle, cartilage-like tissue constructs and components of the human heart (de Melo et al., 2019; Lee et al., 2019; Lee et al., 2020b; Kupfer et al., 2020). On the other hand, cell-laden hydrogels are the most frequently used and attractive choice to mimic the native niche (Mirdamadi et al., 2019; Spencer et al., 2019; Mancha Sanchez et al., 2020). Possible applications of cross-linked hydrogels as bioink of bath for bioprinting in IUA are foreseen for the repair of damaged endometrium, regenerative drugs or cell delivery. However, bioinspired hydrogels cannot become the first-line therapy in endometrial regeneration mainly because, the use of hydrogels as a single without a traditional IUD anti-adhesion barrier is less satisfactory. Increasing the mechanical strength can make hydrogel capable to meet the requirement of a culture system for the formation of normal functional endometrium for embryo implantation. In addition, for the clinically relevant applications, focus is needed toward possible chemical crosslinking and mechanical intigrety to withstand the conventional sterilization methods as terminal sterilization is difficult and time-consuming because of the hydrated nature of hydrogels (Lima et al., 2020). All these questions are still worth the continued efforts in the future.
